# Trench Fever Caused by a Resistant Filterable Virus

**Published:** 1918-09

**Authors:** 


### TRENCH FEVER CAUSED BY A RESISTANT FILTERABLE VIRUS

At the Meeting of the Research Committee of the A. R. C. on June 29th, a report of the progress of the investigations of the Trench Fever Committee was read by its Chairman, Major Richard P. Strong.

The most recent contribution to the progress in medical science announced by this Committee is that Trench Fever has been proved to be caused by a resistant filterable virus. The completed report of the Trench Fever Committee is now being published by the Oxford Press for the American Red Cross in France, and it will soon be ready for distribution. The following is a summary of the report of recent researches on Trench Fever trade at the June meeting of the Research Committee :

" The experimental work undertaken in connection with trench fever has been practically completed. In these investigations 102 different human experiments have been carried out in connection with the study of the transmission of trench fever and its cause. Of these 28 have been in connection with blood transmission, 38 in connection with transmission by lice, 16 in connection with transmission by the urine, feces and sputum, and 20 in connection with the nature of the virus of trench fever. The disease has been produced experimentally by different methods in 62 instances. This is probably the first time in history where an infectious disease has been studied in such a thorough manner on so many human subjects.

The most important facts which have been demonstrated by these investigations are :

1. That trench fever is a specific, infectious disease; that it is not a modified form of typhoid or paratyphoid fever, and is not related, from an etiological standpoint, to these diseases.

2. That the organism causing the disease is a resistant filterable virus.

3. That the virus causing trench fever is present particularly in the plasms of the blood of trench fever cases, and that such plasms will produce the disease on inoculation into healthy individuals.

4. That disease is transmitted naturally by the louse pediculus humanus, Linn. var. corporis, and that this is the important and common means of transmission; that the louse may transmit the disease by its bite alone, the usual manner of infection, or the disease may be produced artificially by scarifying the skin and rubbing in a small amount of the excrement from infected lice.

5. That a man may be entirely free from lice at the time he develops trench fever, the louse that infected him having left him some time previously as its host, that the louse need only remain upon the individual for a short period of time in order to infect him.

6. That the virus of trench fever is also sometimes present in the urine of trench fever cases and occasionally in the sputum, and that disease may be produced in man from the introduction of the virus in the urine or sputum through the scarified or otherwise abraded skin.

7. That since the urine and sometimes the sputum of trench fever patients are infective, these should be sterilized in order to avoid the possibility of accidental infection from them.

8. That in order to prevent trench fever, or limit its spread, and thus save man power for the armies, greater efforts must be made to keep soldiers in general from infestation with lice."

